# Beyond the RNA-dependent function of LncRNA genes

**DOI:** 10.7554/eLife.60583

**Published:** 2020-10-23

**Authors:** Tamer Ali, Phillip Grote

**Affiliations:** 1 Institute of Cardiovascular Regeneration, Centre for Molecular Medicine, Goethe UniversityFrankfurt am MainGermany; 2 Faculty of Science, Benha UniversityBenhaEgypt; Max Planck Institute for Heart and Lung ResearchGermany; Max Planck Institute for Heart and Lung ResearchGermany

**Keywords:** lncRNA, gene regulation, 3D genome

## Abstract

While long non-coding RNA (lncRNA) genes have attracted a lot of attention in the last decade, the focus regarding their mechanisms of action has been primarily on the RNA product of these genes. Recent work on several lncRNAs genes demonstrates that not only is the produced RNA species important, but also that transcription of the lncRNA locus alone can have regulatory functions. Like the functions of lncRNA transcripts, the mechanisms that underlie these genome-based functions are varied. Here we highlight some of these examples and provide an outlook on how the functional mechanisms of a lncRNA gene can be determined.

## LncRNA genes in the genome

**Figure 1.**
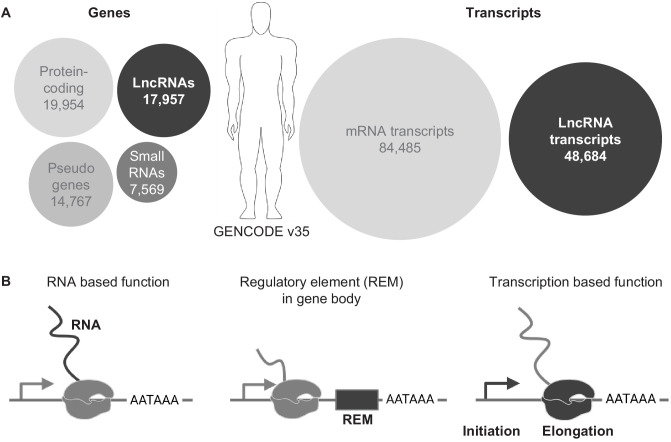
LncRNA genes in the genome. (**A**) Overview of genes and transcript numbers in the human genome (GENCODE v35). Circle area represents relative quantities. (**B**) Schematics of three possible functional properties of lncRNA loci.

The complex genome of eukaryotes is pervasively transcribed and efforts to comprehensively define all transcripts have led to the idea that about half of the genome can be transcribed into RNA in an individual cell (Djebali et al., 2012). The units that produce RNAs – the genes - can roughly be categorized into the two main biotypes: protein-coding genes (PCGs) and non-protein-coding genes (NCGs). The largest and most coherent category is the PCG, which encodes RNAs that serve as the template for all the peptides and proteins in the cell. The NCG category is a highly heterogenous collection and can be sub-grouped into small ncRNA (non-coding RNA) and long ncRNA (lncRNA) genes, where the term long refers to the arbitrary length of 200 nucleotides or longer. In particular, the lncRNA genes have attracted a lot of attention in recent years due to their wide range of action and mostly unexplored functions. While their number was overestimated after their initial discovery, similar to the overestimation of the number of PCGs at the beginning of the human genome project (Lander et al., 2001), current and careful curation projects, such as the GENCODE and FANTOM projects, list 17,957 and 27,919 lncRNA genes, respectively (Figure 1A), in their most recent data releases of the human genome (Frankish et al., 2019; Hon et al., 2017). Hence, the number of lncRNA genes are in the same range, or even a bit higher, than the number of PCGs (19,954). In the future, this currently very heterogeneous class of NCGs may be sub-categorized further into more specific biotypes.

Currently, three major functional principles can be assigned to lncRNA loci (Figure 1B): (1) either the RNA is the functional biomolecule and interacts with other components in the cell, for example DNA, proteins or RNAs, (2) a gene regulatory element is embedded in the transcription body of a lncRNA gene and the activity of the lncRNA gene directs the activity of the regulatory element or (3) the process of transcription influences genome and thereby gene activity. A lncRNA locus can haveone of these functions or a mixture of them (Yin et al., 2015). In this review we will focus on the latter two functional lncRNA properties, in which the RNA is, at least partially dispensable for the lncRNA gene function.

### The transcription of genes

**Figure 2.**
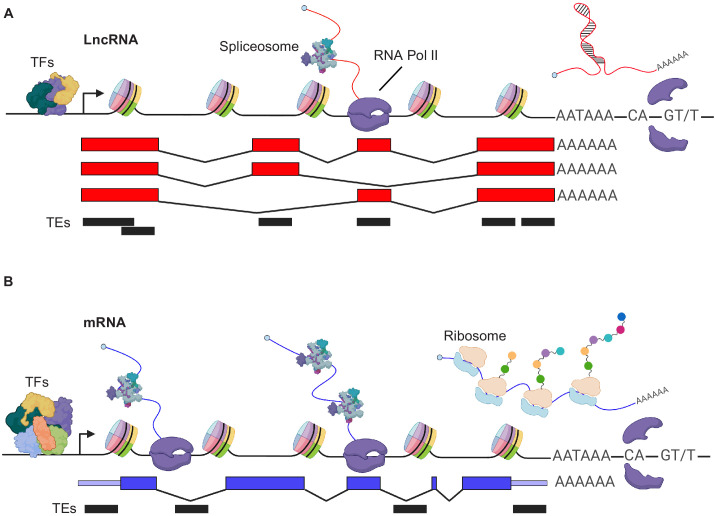
Distinguishing features of transcript generation of PCGs and lncRNAs (**A**) LncRNA and (**B**) mRNAs: lncRNA genes are lowly expressed as fewer transcription factors (TFs) bind the promoter. In addition, lncRNA TSS, exon and/or pA site more often associate with transposable elements (TEs), while TEs contribute mostly to UTRs and/or introns of mRNAs. In addition, mRNAs are more efficiently spliced.

The generation of RNA using the genome as a template, or the process of transcription, depends on certain functional genomic elements (Figure 2). The core element of a gene that initiates the production of an RNA is the promoter. A GC-rich element that is accessible (open chromatin) will attract the polymerase machinery and general transcription factors (TFs). This minimal core element serves as a core promoter and can be sufficient to initiate transcription (Deaton and Bird, 2011). Transcription of RNA starts at the transcriptional start site (TSS), which is located within the core promoter. Like PCGs, most lncRNAs are transcribed by POL II (RNA polymerase 2, a multiprotein complex), but are more tissue-specific compared to PCGs (for review see Ransohoff et al., 2018). Both biotypes (PCGs and lncRNAs) have conserved core promoter sequences with fewer overlapping TF binding motifs in lncRNA promoters, resulting in an overall lower expression level compared to PCGs (Figure 2; Mattioli et al., 2019). Thus, the architecture of the core promoter is the first player that defines the degree of lncRNA expression (Batut and Gingeras, 2017; Mattioli et al., 2019). The second important element that influences the transcription of genes are enhancers, which are *cis*-regulatory elements that can either have a positive or a negative (which are then often referred to as repressors) impact on their target genes. Consequently, enhancers are genomic regions that encode binding sites for sequence-specific activator or repressor TFs. These elements often confer specificity in spatiotemporal expression. Many lncRNAs can also be generated from such enhancer elements, which contributes to their overall more tissue-specific expression when compared to PCGs (Mattioli et al., 2019).

The core promoter initiates transcription and thereby the generation of an RNA that may or may not be further diversified by splicing (Figure 2). This depends on whether splice sites are present between the promoter and the transcription termination element, the polyadenylation signal (pA). The mechanism of PCG and lncRNA splicing is similar, although the splicing efficiency of lncRNAs is lower than PCGs, likely due to the loss of proximal RNA POL II phosphorylation over 5’ splice sites (Krchnáková et al., 2019). In addition, lncRNAs show signs of co-transcriptional cleavage and premature termination with Thr4p PolII enriched over the entire lncRNA body (Schlackow et al., 2017). At some point the transcriptional machinery will run into a termination signal, a DNA sequence element consisting of AATAAA and downstream GU (or U)-rich motifs (Eaton et al., 2020). These elements are ubiquitously present in the genome. In humans, one can find 569,005 elements that meet the criterion of a pA signal (301,001 in mouse and 20,931 in *C. elegans*) (Herrmann et al., 2020). Moreover, this high number likely ensures successful termination of transcription (Eaton and West, 2020).

Another class of genetic elements that play an important role for gene and genome activity are transposable elements (TEs) (for review see Chuong et al., 2017). These mobile genomic elements make up more than 44% of the human genome (Lander et al., 2001) and attracted attention as important regulators of gene and genome activity (Bourque et al., 2018). In this respect, TEs are an important component of lncRNA biology as well (Figure 2A). Approximately, 75% of lncRNA transcripts contain sequence elements from TEs (Kapusta et al., 2013) and some of them represent important sequence elements to direct lncRNA localization (Lubelsky and Ulitsky, 2018). In addition, 25% of TEs are found to overlap with TSS and pA signals of lncRNA genes (Kapusta et al., 2013). Hence, they are an important driving force of lncRNA expression. One recent example is the primate-specific lncRNA *XACT* (Table 1), which has been shown to protect the active X chromosome from being silenced (antagonizing *XIST* lncRNA effect) and whose sequence contains elements derived from a TE (Casanova et al., 2019). Interestingly, *XACT* lncRNA is also regulated by a TE-derived enhancer element that harbors pioneer pluripotency factor binding sites. This exemplifies that TEs containing embedded TF motifs can direct tissue-specific expression when they insert next to a promoter element. Several other TE-derived lncRNAs are described elsewhere (Kapusta et al., 2013).

**Table 1.** Selection of lncRNA genes with RNA independent function.

LncRNA	Relative location of respective TSSs target gene	Literature	Mode of action	
Regulatory element located within the transcription unit				
*Haunt (Halr1)*	40 kb downstream of *HOXA*	Yin et al., 2015	Activation of HOXA	
*Lockd*	4 kb downstream of *Cdkn1b*	Paralkar et al., 2016	Positive regulation of *Cdkn1b* via loop formation	
*Meteor*	80 kb upstream of *Eomes*	Alexanian et al., 2017	Positive licensing of *Eomes *expression	
*ThymoD*	844 kb downstream of *Bcl11b*	Isoda et al., 2017	DNA methylation, CTCF-binding	
*Pcdhα-as*	*Pcdhα*	Canzio et al., 2019	DNA methylation, CTCF-binding	
*GAL10-ncRNA*	*GAL10* antisense transcript	Houseley et al., 2008	*GAL10* promoter acetylation	
*AIRN*	28 kb Antisense to *Igfr2*	Latos et al., 2012	Promoter methylation	
*Upperhand (Hand2os1)*	0,1 kb upstream of *Hand2*	Anderson et al., 2016; Han et al., 2019	Promotes enhancer accessibility for *Hand2* activation	
Activity exerted by transcription initiation or elongation				
*Ftx*	140 kb upstream of *Xist*	Furlan et al., 2018	*Xist* activation independent of *Ftx* RNA	
*Chaserr*	16 kb upstream of *Chd2*	Rom et al., 2019	Negative regulation of *Chd2*	
*PVT1*	52 kb downstream of *Myc*	Cho et al., 2018	Enhancer boundary element	
*Handsdown* (*Handlr*)	11 kb downstream of *Hand2*	George et al., 2019; Ritter et al., 2019	Transcriptional elongation-based enhancer shielding

In summary, the genome stores the information required to generate the RNAs that are necessary for a cell’s proper function, whether the RNA is protein-coding or not. An elaborate machinery is established that controls the specific activation of genes and whole genomic regions via positive or negative mechanisms. These regulatory mechanisms require energy investment from the cell. It is conceivable that sometimes it can be ‘cheaper’ for a cell to let spurious transcription of non-harmful transcripts occur, might they be coding or non-coding, than to invest energy in silencing all of these transcriptionally active sites.

### Layers of gene regulation

The expression of genes and whole genomic regions is controlled by several layers of regulation. In addition to the genomic elements described above, DNA is packed with histone proteins into chromatin. These protein components can be modified to act as signaling centers for the transcription machinery (for review see Talbert et al., 2019). In addition, the proteins of the nucleus also regulate the 3D arrangement of genomic DNA in such a way that functionally connected elements of gene regulation come together. In short, each chromosome is composed of sub-megabase units known as topologically associated domains (TADs), the structural and functional unit of the chromosome (for review see Szabo et al., 2019). Such genome arrangements can allow for promoter-enhancer contacts and organize functionally dependent regulatory elements together (Hnisz et al., 2017). The major factors that regulate this organization are CTCF (CCCTC-binding TF) and the cohesin complex (Ali et al., 2016; Rao et al., 2017). CTCF binding frequently co-localizes and interacts with the cohesin complex at TAD borders (Li et al., 2020). Indeed, elimination of cohesin dissolves all chromatin TADs even in the presence of CTCF (Rao et al., 2017). Interestingly, disruption of the TADs either by removal of CTCF or cohesin results in unexpected mild effects on gene expression (Nora et al., 2017; Rao et al., 2017). While it has been accepted that gene expression and 3D genome folding are correlated, their functional relevance is still to be elucidated (Ibrahim and Mundlos, 2020).

All of these enhancers and genome organizing regions must be functionally regulated to accurately control gene and genome activity. As many such regulatory sites are associated with lncRNAs, these lncRNA loci might be important functional support elements. The process of transcription can assist in reorganizing chromatin marks (van Steensel and Furlong, 2019), allowing regions to be accessible for other factors or prevent others by diverting/directing the transcription machinery to nearby genes.

### Current annotations in the database are a work-in-progress

Current annotations of genomic databases categorize genes according to various criteria. One that appears, on the surface, to be very simple is the separation of protein-coding genes (PCGs) and non-protein-coding genes (NCGs). It was already found some time ago that RNAs originating from NCGs do actually associate with ribosomes, the machinery that translates mRNAs into proteins (Ingolia et al., 2011; van Heesch et al., 2014). This association is not surprising, as the ribosomes function is to bind RNAs in the cytosol and attempt to translate it into a peptide or protein. However, just because an RNA is bound to a ribosome does not mean it is translated and even if translated, the pure presence of a peptide does not prove a function of this peptide. In more recent in-depth studies, it was found that some lncRNAs do produce peptides and that some of these peptides are even functional (Chen et al., 2020; Ji et al., 2015; van Heesch et al., 2019), including within 5’ and 3’ untranslated regions (UTR) of mRNAs. Hence, until databases are updated with suitable information that incorporates the presence of peptides derived from expressed RNAs, a peptide coding probability always must be taken into consideration when studying lncRNA function. Equally important, many PCG or NCGs have a high number of splice variants, some of which might encode a peptide and others not.

The revolution of high-throughput sequencing of fragmented cDNA libraries revealed the complexity of expression from the genome. Enrichment of lowly expressed transcripts and subsequent sequence analysis identified an even more complex pattern of splice variants (Mercer et al., 2012). However, these analyses relied on the sequencing of fragmented cDNA libraries and subsequent reconstruction of the transcriptome to a reference genome. The most recent generation of long read sequencers, such as the PacBio or the Nanopore systems, allows the direct analysis of RNAs and eliminates the intermediate step of a fragmented cDNA library. Capturing lncRNA genes specifically and resequencing by Long-read platform (known as Capture Long Sequence or CLS) determined the full variety of splice variants of the mammalian transcriptome (Lagarde et al., 2017). The advantage of this technology is the capability to precisely determine 5’ and 3’ ends and, ideally, all splice variants of a transcript. For example, the estimated mean number of exons per lncRNA using CLS was 4.27 compared to 3.59 measured by short-reads RNA-seq method (Lagarde et al., 2017). While this approach doesn’t eliminate the necessity to carefully determine the splice variants from a lncRNA locus entirely, it does provide a very good starting point for detailed analysis. In particular, when CLS data are not available for your locus-of-interest or your tissue-of-interest, one should determine the full transcript length, splice variants and regulatory elements of the lncRNA-of-interest. Only then can a successful strategy to study the lncRNA be initiated.

### Gene regulation by lncRNA genes – regulatory elements within the transcription unit

Surveying the chromatin and DNA modification landscape led to the annotation of potential regulatory regions across the genome and sometimes even for specific tissues and cell types. Regulatory elements, whether they are promoters or other regulatory elements, can be found within or far away from the transcription unit of a gene. The occurrence of such a regulatory element within a transcription unit, for example of a lncRNA gene, can indicate that the function of this element might be affected by its activity.

One interesting lncRNA gene example that reflects the duality of lncRNA genes with respect to their RNA-based mechanism on one side, and an enhancer element on the other side, is *Haunt*. While the RNA of *Haunt* is thought to be required for negative regulation of *HoxA*, the *Haunt* locus contains regulatory elements to activate the *HoxA* locus during *in vitro* differentiation of pluripotent stem cells (Yin et al., 2015). While it is shown that these enhancers can interact with *HoxA* directly, the elements are not further defined nor how their function might depend on *Haunt* transcriptional activity.

A similar early example of a lncRNA locus that contains a regulatory element within its transcription unit is the *Lockd* lncRNA locus, which regulates its *cis* gene *Cdkn1b*. The deletion of the entire locus of *Lockd*, including TSS upstream elements, leads to a reduction of *Cdkn1b* expression (Paralkar et al., 2016). While the 5’ genomic region of *Lockd* interacts genomically with the promoter of *Cdkn1b*, this interaction is not altered if the transcription of *Lockd* is depleted by a pA signal inserted into the first exon of *Lockd*. Thus, the genomic locus itself is important as an regulatory element rather than its transcriptional activity.

Even if a specific regulatory element cannot be defined, careful analysis and genetic dissection of a lncRNA can point toward such a regulatory principle. The TSS of the *Meteor* lncRNA locus is important to license its *cis*-located gene *Eomes* for activation in the mesendoderm (Alexanian et al., 2017). The lack of *Meteor* expression by TSS deletion causes the loss of *Eomes* activation during mesendoderm differentiation of mouse ESCs. Decreasing levels of *Meteor* RNA during this process did not alter expression of downstream genes, arguing against an RNA-based function of *Meteor*. Interestingly, endogenous activation of *Meteor* is not only licensing *Eomes* gene activation, but other cardiac mesodermal genes as well. Moreover, transcriptional inhibition of *Meteor* using a polyadenylation element insertion downstream of the *Meteor* TSS does not cause the *Eomes* gene to be silenced during mesendoderm differentiation (Alexanian et al., 2017; Engreitz et al., 2016). This finding argues against a transcription-based mechanism of *Meteor* and suggests that the genomic locus *Meteor* harbors important regulatory elements to render the *cis*-located *Eomes* gene activatable during differentiation.

**Figure 3.**
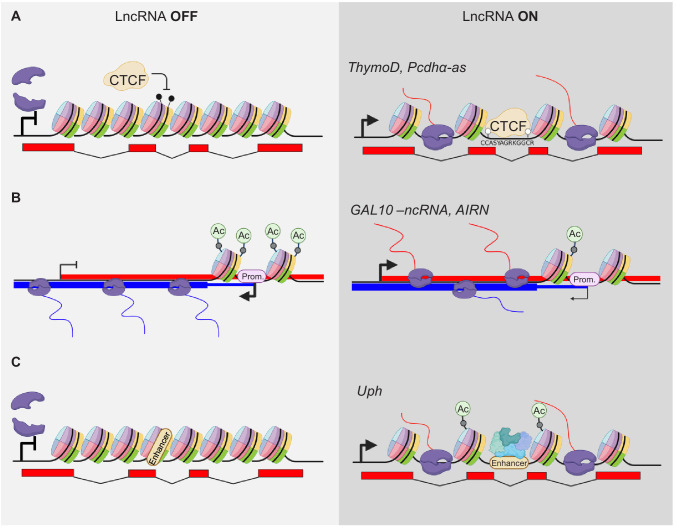
Modulation of gene expression by lncRNA transcription. (**A**) Transcriptional activity modulates DNA methylation and thereby alters occupation of DNA binding factors within the gene body, for example CTCF. The POL2 complex is indicated in violet. Black drumsticks indicate methylated CpGs, white drumstick non-methylated CpGs. (**B**) LncRNA expression alters promoter (Prom.) activity by modifying e.g. acetylation of histones at TSS sites. (**C**) Transcription elongation can activate poised enhancers within their gene body (only acetylation shown).

An excellent example of a lncRNA with a defined regulatory element within transcription unit is the *ThymoD* lncRNA locus. Its transcription prevents methylation of a CTCF-binding site located within its transcriptional unit (Isoda et al., 2017; Figure 3A). The binding of CTCF allows looping of the *Bcl11b* transcription unit in the same domain as activating regions of *Bcl11b*. This activation is lost when the transcription of *ThymoD* is blocked by insertion of a pA signal after exon two and before the CTCF-binding site and, consequentially, the CTCF-binding site is methylated (Figure 3A). Therefore, the transcriptional activity has an indirect, structural effect on the regulation of *Bcl11b* while the *ThymoD* RNA is dispensable.

A more complex situation of several antisense transcripts regulating their *cis* gene is the Protocadherin alpha (*Pcdhα*) cluster. The variable, stochastic expression from several Protocadherin clusters provide cell-surface proteins for cellular identity recognition in the neuronal system to allow dendrites and axons to distinguish from self and other neurons. This stochastic expression is partly regulated by a distal enhancer region. The cluster of *Pcdhα* produces three distinct variants from three alternative TSSs to achieve stochastic expression of splice variants from this cluster. The first exon of each of these variants contains an antisense lncRNA transcript (*Pcdhα-as*) (Canzio et al., 2019). The expression of the lncRNAs precedes the expression of the PCGs and positively regulates the most nearby PCG expression. Mechanistically, the *Pcdhα* lncRNAs act similar to the *ThymoD* lncRNA (above) (Figure 3A). Expression of the *Pcdhα-as* variants leads to the demethylation of a CTCF-binding site in the region upstream of the *Pcdhα* PCG, thereby allowing for a stable loop formation with the distal enhancer region and a positive effect on the PCGs expression.

There are also examples of lncRNA genes that reside within a different transcriptional entity from *cis* target genes. Here, it is even more conceivable that their activity has an impact on the gene they are embedded in. One of the first examples was a ncRNA within the *GAL10* gene cluster in yeast *Saccharomyces cerevisiae*. Under 0% galactose, the TF Reb1 binds to the promoter region of *GAL10-ncRNA* antisense to *GAL10* and fully activates its expression (Houseley et al., 2008). The transcriptional unit of *GAL10-ncRNA* overlaps with the TSS of *GAL10* and *GAL1*, leading to inhibition of the *GAL10* and *GAL1* gene by promoting high levels of H3K36me3 methylation and hypoacetylation at the *GAL10* and *GAL1* promoters. Addition of galactose to the growth medium blocks *GAL10-ncRNA* expression and hyperacetylation of the *GAL10* and *GAL1* promoters, leading to expression of genes that encode galactose fermenting proteins (Figure 3B).

A similar principle was shown in higher eukaryotes at the *AIRN* (antisense *Igf2r* RNA non-coding) locus. The TSS of the lncRNA *AIRN* is located in the second intron of the *Igf2r* PCG and *AIRN* is transcribed antisense to *Ifg2r*. Transcription of *AIRN* negatively regulates *Igfr2* (Santoro et al., 2013). When transcription of AIRN is blocked by a polyA insertion before the promoter of *Igf2r,* this negative regulation is abolished (Figure 3B). However, if the same pA is inserted after the promotor of *Igf2r*, this negative regulatory effect on *Igf2r* is not observed (Latos et al., 2012). These findings support the hypothesis that the transcription of *AIRN*, and not the RNA product itself, is important for the transcriptional regulation of the *Igfr2*.

A lncRNA gene transcription that influences an enhancer is *Upperhand*, which is divergently expressed from the *Hand2* protein-coding gene (Anderson et al., 2016). Loss of *Upperhand* transcription leads to a loss of histone acetylation upstream of *Hand2*, including at the cardiac enhancer. As a result, binding of GATA4 to its previously defined enhancer (McFadden et al., 2000) is reduced, and *Hand2* expression in the heart is reduced as well. Hence, the Upperhand loss-of-function phenotype is similar to cardiac loss of *Hand2* (Figure 3A). Additional mutants of *Upperhand* draw a more complicated picture of the role of *Upperhand* in activating *Hand2*. A complete deletion of the *Upperhand* transcription unit that encompasses all known regulatory regions of the *Hand2* gene as well, causes loss of *Hand2* 5’UTR expression (Han et al., 2019). These findings assert the presence of important *Hand2* activating genetic elements directly upstream of its TSS, independently of any RNA originating from this region. However, a promoter deletion of *Upperhand* causes a loss of its RNA while leaving all other elements in that region intact, but no effect on *Hand2* expression was observed in this case. Furthermore, a deletion of the last two exons from *Upperhand* has a slight effect on *Hand2* expression. There might be so far uncharacterized enhancer elements in the genomic region of these two exons and their deletion may influence *Hand2* expression. In addition, although the *Upperhand* RNA is suggested to be not required for its *in vivo* function, the RNA generates peptides that might be functional (van Heesch et al., 2019). These somehow conflicting results underline the complexity of regulation of the *Hand2* gene.

These examples highlight the importance of taking a careful look at the whole lncRNA locus that produces an RNA. The occurrence of an annotated regulatory element or the occupation of a genome regulating factor such as CTCF within the transcription unit can be an important indication to look for a genomic function of a lncRNA.

### Gene regulation by lncRNA genes – the act of transcription is functional

The absence of a regulatory element within the transcription unit could be due to incomplete annotation or a yet unknown factor which binds there, or the act of transcription initiation or transcriptional elongation is important for the function of a lncRNA locus.

**Figure 4.**
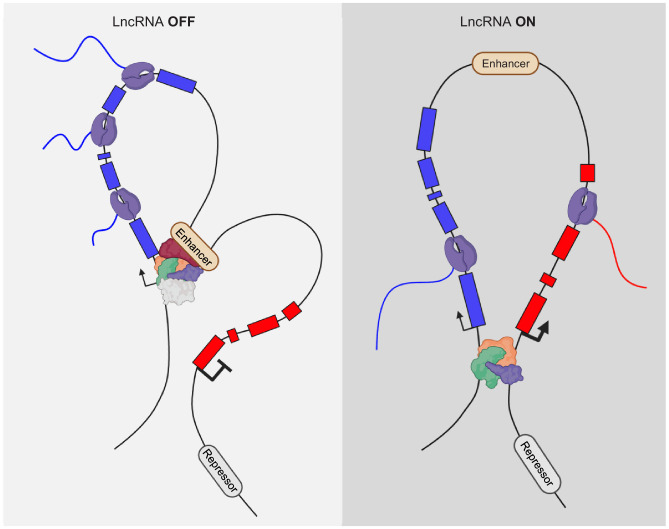
Alteration of genome interactions by lncRNA activity. DNA:DNA contacts can change upon transcriptional activity of nearby, *cis* located lncRNA genes.

One example of such a regulation principle comes from work on the *XIST* lncRNA, which is one of the original lncRNAs that has been extensively studied (Brockdorff et al., 1992). While XIST acts via the produced RNA (Brannan et al., 1990; Brown et al., 1992), the regulation of *XIST,* at least in part, does not. The *XIST* lncRNA locus is flanked by many lncRNAs, and one of them is the *Ftx* locus found 140 kb upstream of *Xist* (Chureau et al., 2002). It was initially proposed that the *Ftx* RNA functions to regulate *XIST* (Chureau et al., 2011). However, detailed analysis uncovered that the transcription of *Ftx*, and not the produced RNA, is important to regulate *Xist* (Furlan et al., 2018). Knockdown of *Ftx* RNA does not cause a loss of *Xist* expression, but deletion of the promoter of *Ftx*, and the consequential loss of *Ftx* transcription, causes the loss of *Xist* expression. CRISPRi of *Ftx* similarly causes loss of *Xist* expression, suggesting that transcription of *Ftx* is the positive regulator of *Xist* expression. One possibility is that 3D genome architecture can be changed due to the transcriptional activity of a genomic locus (Figure 4). Strikingly, the promoter of *Xist* and *Ftx* are flanked by CTCF-occupied sites. However, deleting the CTCF-binding sites alone at the *Ftx* promoter has no effect on the expression level of *Xist*, arguing that genome folding induced by *Ftx* activity does not involve CTCF-binding.

Another good example is the *Chaserr* lncRNA locus, which lies 16 kb upstream of the *Chd2* protein- coding gene (Rom et al., 2019). Although, knock-down of *Chaserr* RNA does cause a slight increase in *Chd2* expression, additional lines of evidence infer that the transcription of the lncRNA gene is likely the most important function of *Chaserr* in regulating *Chd2* (Figure 4). In addition, the promoter of *Chaserr* interacts with the *Chd2* promoter in chromosome conformation capture analysis. Upon deletion of the *Chaserr* promoter region, the *Chd2* promoter increasingly interacts with other enhancer elements upstream. In contrast, if the gene body of *Chaserr* is deleted, leaving the promoter intact, these changes in enhancer/*Chd2*-promoter contacts are not observed. A plausible explanation is that the transcription initiation activity rather than the transcription elongation is important for regulation of *Chd2* by *Chaserr*.

Similarly, transcription initiation is important for the *PVT1* lncRNA locus. The *Pvt-1* lncRNA was originally discovered as a genomic translocation that causes the activation of the *Myc* oncogene (Adams and Cory, 1985). Initially, it was suggested that miRNAs embedded in the lncRNA transcript of *PVT1* are important for regulation of target genes (Wang et al., 2019). It turns out that *PVT1* transcription has an RNA-independent function as well. The *PVT1* locus encodes several transcripts with alternative start sites. The activity of its major TSS serves as a boundary element to shield the *MYC* promoter from over-activation by an enhancer located within the transcriptional unit of *PVT1* (Cho et al., 2018; Figure 4). The transcriptional activity is important for this shielding capacity, but not the elongation of the transcription (Figure 4). This does not mean that the miRNAs produced by *PVT1* do not serve a function, but it seems the major activity of the *PVT1* lncRNA, and its effect on *MYC* is conveyed by the transcriptional activation of *PVT1*.

In addition to the *Upperhand* lncRNA upstream of *Hand2* (see above), there are *Hand2-*regulating lncRNA loci downstream of *Hand2*. We initially characterized this locus and termed it *Handsdown*, due its location downstream of *Hand2*. The *Handsdown* locus is expressed in the same tissues as *Hand2* but is most significantly expressed in the developing heart. We have shown that transcription of *Handsdown* is important to negatively regulate the expression of *Hand2* (Figure 4). Moreover, the HAND2 TF binds two distinct sites around the TSS of *Handsdown* in the developing E9.5 heart (Laurent et al., 2017). This suggests that HAND2 activates its own suppressor region in a negative feedback loop to control its dosage. However, deletion of the TSS region of *Handsdown*, including only one of the HAND2 occupied sites, does not result in the expected upregulation of *Hand2* (George et al., 2019). Multiple, potential TSS regions are present in at the 5’ region of *Handsdown* and the deletion of one or the major TSS can lead to the appearance of alternate transcripts (Lavalou et al., 2019). Therefore, it is plausible that the second HAND2 occupied site may be sufficient to instruct the transcription of an alternate *Handsdown* transcript. Hence, as long as transcriptional activity is present in the *Handsdown* region, *Hand2* can be negatively regulated and its expression level adjusted. The dosage of *Hand2* is particularly important as loss of one copy of the *Hand2* gene, as well as the gain of an additional copy of the *Hand2* gene, causes malformations during development (Tamura et al., 2014). In addition to these lncRNA loci flanking the *Hand2* gene, additional putative enhancers are predicted up- and downstream of *Hand2*, underlining the complex regulome of this important gene in development.

While functions of lncRNAs on the transcript level are becoming increasingly understood, elucidating mechanisms of how such loci, whose function is based on the transcriptional level, exhibit their effect (Table 1) is still in its infancy. While this list is not saturated, the number of lncRNAs that at least partially act by such a mechanism will increase in the future. One very promising model of how they may act are functional microdomains. In such a scenario, these microdomains promote the co-operativity between interacting components such as TFs, co-factors, chromatin regulators, RNA polymerase II, and non-coding RNA, thereby governing basic processes of gene regulation. Such microdomains are favorably formed by super-enhancers that also often generate an RNA, but function on the transcriptional level. Hence, transcriptional activity itself can influence chromatin accessibility, DNA methylation, histone modification, and higher order chromatin structure.

## Outlook

A core question for the near future is to define which of the lncRNA loci are functional on the transcript (RNA) level or on the transcriptional (genome) level and which loci may function on both levels.

The widespread use of the CRISPR toolbox does allow for the generation of targeted genomic modifications to dissect the mode-of-action of a lncRNA locus. With CRISPR/Cas9 deletions even in the mega-base range being feasible, the deletion of the entire transcription unit will allow one to determine if a lncRNA locus is functional at all (Barutcu et al., 2018; Kraft et al., 2015). This crude approach eliminates any transcript coming from the locus, also eliminating the possibility that degradation of any residual transcript does cause any effect by, for example, genetic compensation (El-Brolosy et al., 2019). Simply put: if the removal of a complete lncRNA locus does not result in even subtle effects on gene expression, this locus can be marked non-functional, at least in the analyzed biological system. Subsequently, the promoter encompassing the TSS can be removed to eliminate any transcriptional initiation of the transcript. A similar result can be achieved to use the CRISPRi (Ferreira et al., 2018) system to shut down the locus without removing any parts of the genome. It has to be kept in mind, that removing a TSS might trigger the emergence of new transcripts from secondary TSS-like sites in the vicinity (Lavalou et al., 2019). It is therefore important to evaluate this possibility and verify that no ‘novel’ transcripts arise. To interfere with transcriptional elongation and also study effects in regulatory elements within a gene body, the transcription can be terminated using a strong transcriptional stop signal. To allow for efficient targeting using the CRISPR toolbox, a short and powerful pA signal is preferred (Ballarino et al., 2018; Lavalou et al., 2019). In combination with an endogenous CRISPRa (Konermann et al., 2015) system, this meddling with the lncRNA does allow for a detailed assessment of its function. In particular, subsequent removal of parts of a lncRNA locus, for example, whole exons or potential regulatory elements within the transcription unit will allow one to define the functional elements on either the RNA or the locus.

The powerful tool of antisense oligonucleotide (ASO)-assisted knock-down of RNA can now help allow for a detailed assessment of RNA vs transcription-based function. Until now, if a lncRNA transcript was inhibited on the RNA level by antisense oligo methods, siRNA or locked nucleic acid (LNA)-based ASO, it was assumed that the RNA, rather than its transcription was important for the resulting phenotype. Initially, ASOs, which employ the endogenous RNAseH enzyme for target RNA degradation (Grünweller et al., 2003), were the method of choice, as they can target nuclear and cytosolic RNA similarly well. However, it turns out one must be a bit more cautious with this assumption. Several recent publications demonstrate that ASOs that target the 5’ end of an RNA can do this even on nascent RNA that is in the process of being transcribed (Eaton et al., 2020; Lai et al., 2020; Lee and Mendell, 2020). This premature cleavage of RNA leads to the recruitment of XRN2 and employs the torpedo mechanism to evict the POL II transcription machinery prematurely. Hence, a 5’ directed ASO mimics the loss of transcriptional elongation and may lead to confusion about potential lncRNA mechanistic function. To validate an RNA-based mechanism by ASOs, it is preferred to target the 3’ end of the RNA-of-interest. But, more importantly, this mechanism opens the possibility to target lncRNA (or any other locus) whose mechanism requires transcriptional elongation until the endogenous transcriptional termination site, independent of whether the RNA is functional. Carefully designed experiments can increase our understanding of which lncRNA functions are important for gene regulation, which can be beneficial in studying human disease involving dysregulation by such loci. Furthermore, this extends the repertoire of loci that can be targeted for studying the lncRNA genes and their therapeutic use. Now, not only genes that produce a functional RNA can be therapeutically targeted, but also any gene or regulatory locus that generates an RNA and has a gene or genome regulatory function via transcriptional elongation, *per se*, is amenable to ASO targeting.
